# Risk Factors and Imaging Mechanisms of Fatigue After Mild Ischemic Stroke: An Exploratory Study From a Single Chinese Center

**DOI:** 10.3389/fneur.2021.649021

**Published:** 2021-05-25

**Authors:** Xiaoxiao Zhang, Hongjuan Fang, Ding Ma, Yunyun Duan, Zhaozhao Wang, Ning Zhang, Chunxue Wang

**Affiliations:** ^1^Department of Anesthesiology, Beijing Tiantan Hospital, Capital Medical University, Beijing, China; ^2^Department of Endocrinology, Beijing Tiantan Hospital, Capital Medical University, Beijing, China; ^3^Department of Radiology, Beijing Tiantan Hospital, Capital Medical University, Beijing, China; ^4^Department of Radiology, Beijing Friendship Hospital, Capital Medical University, Beijing, China; ^5^Department of Neuropsychiatry and Behavioral Neurology and Clinical Psychology, Beijing Tiantan Hospital, Capital Medical University, Beijing, China; ^6^China National Clinical Research Center for Neurological Diseases, Beijing Tiantan Hospital, Capital Medical University, Beijing, China; ^7^Collaborative Innovation Center for Brain Disorders, Beijing Institute of Brain Disorders, Capital Medical University, Beijing, China

**Keywords:** mild ischemic stroke, biomarkers, risk factors, imaging mechanism, post-stroke fatigue

## Abstract

**Objective:** To explore the biochemical risk factors and imaging mechanisms of post fatigue after mild ischemic stroke among a Chinese population.

**Methods:** Forty consecutive patients with mild ischemic stroke within onset of 14 ± 2 days were enrolled between March and June 2018. The clinical information, scale data, biomarkers in peripheral venous blood, and imaging data during hospitalization and follow-up period were collected.

**Results:** Patient age (range 34–78) was positively correlated with the prevalence of fatigue (*p* = 0.009). Both blood norepinephrine and serotonin levels during hospitalization were negatively correlated to the prevalence of post-stroke fatigue (model 1 *p* = 0.009 and model 2 *P* = 0.043, respectively). Infarct of right cerebral hemisphere is positively correlated with the occurrence of fatigue after mild ischemic stroke (*p* = 0.020). Compared to non-fatigue patients, amplitude of low-frequency fluctuation (ALFF) was lower in several areas of brain in stroke patients with fatigue, including the right orbital inferior frontal, right inner orbital frontal, right frontal, right triangular frontal inferior, right anterior and lateral cingulate, and right medial frontal gyruses. Analysis of the difference in functional connectivity between the fatigue and non-fatigue groups found no cluster.

**Conclusions:** Frontal lobe-related neural pathways may play an essential role in the regulation of fatigue after mild ischemic stroke. Abnormal neural circuits may reduce the levels of neurotransmitters such as serotonin and norepinephrine and lead to post-stroke fatigue.

## Introduction

Stroke is one of the three leading causes of death and the third primary cause of disability worldwide. Post-stroke fatigue is a multi-dimensional emotional and perceptual experience (1), with a high prevalence and a wide range of influences (2). The health hazards of early fatigue and subjective mental fatigue have been overlooked for a long time. Only in recent years, researchers have found that post-stroke fatigue is the main sequelae of stroke (2–6). Most severe stroke patients are disabled and it is hard to accurately evaluate the symptom of post-stroke fatigue. Compared to severe stroke, patients with mild ischemic stroke generally show no obvious motion disability. However, many studies have shown that fatigue significantly lowers the life quality in survivors of mild ischemic stroke and is also a predictor of death after stroke (1, 7–9), which make such patients an ideal candidate for post-stroke fatigue study. Effective diagnosis and treatment of post-stroke fatigue can improve the quality of life for patients with mild ischemic stroke (10–13). In this consideration, it is crucial to understand the pathogenesis of post-stroke fatigue.

The pathogenesis of fatigue after mild ischemic stroke is highly complicated, and there have been limited studies on its biological and imaging mechanisms. Post-stroke fatigue has been confused with post-stroke depression for a long time. In recent years, it has been considered as two different but similar diseases. Although the occurrence of post-stroke depression is known to be related to monoamine neurotransmitters (norepinephrine, serotonin, dopamine), it is unclear if this association is seen in post-stroke fatigue. In a study of neuroanatomical pathway of monoamine neurotransmitter imbalance after stroke, Hama and associates pointed out that the monoamine neural network can be divided into catecholamine and serotonin, which are anatomically and functionally related, and may affect the occurrence of fatigue and emotional disorder after stroke (14). In the present study, we choose these three kinds of monoamine biomarkers to explore the possible mechanisms of post-stroke fatigue.

In the non-stroke fatigue population, it is found that central fatigue may be caused by the failure of integration of marginal input and motor function in the basal ganglia, which affects the striatum thalamus frontal cortex system (15). The relationship between the occurrence of post-stroke fatigue and stroke characteristics (such as its location, type, and neurological deficit) is still unclear (16). Therefore, this study used functional magnetic resonance imaging (fMRI) to explore whether there is abnormal neuronal activity (decrease or increase) in patients with post-stroke fatigue, and whether there is interruption in brain network connection that affects the release of related neurotransmitters.

## Methods

### Patient Population

**Figure 1:**
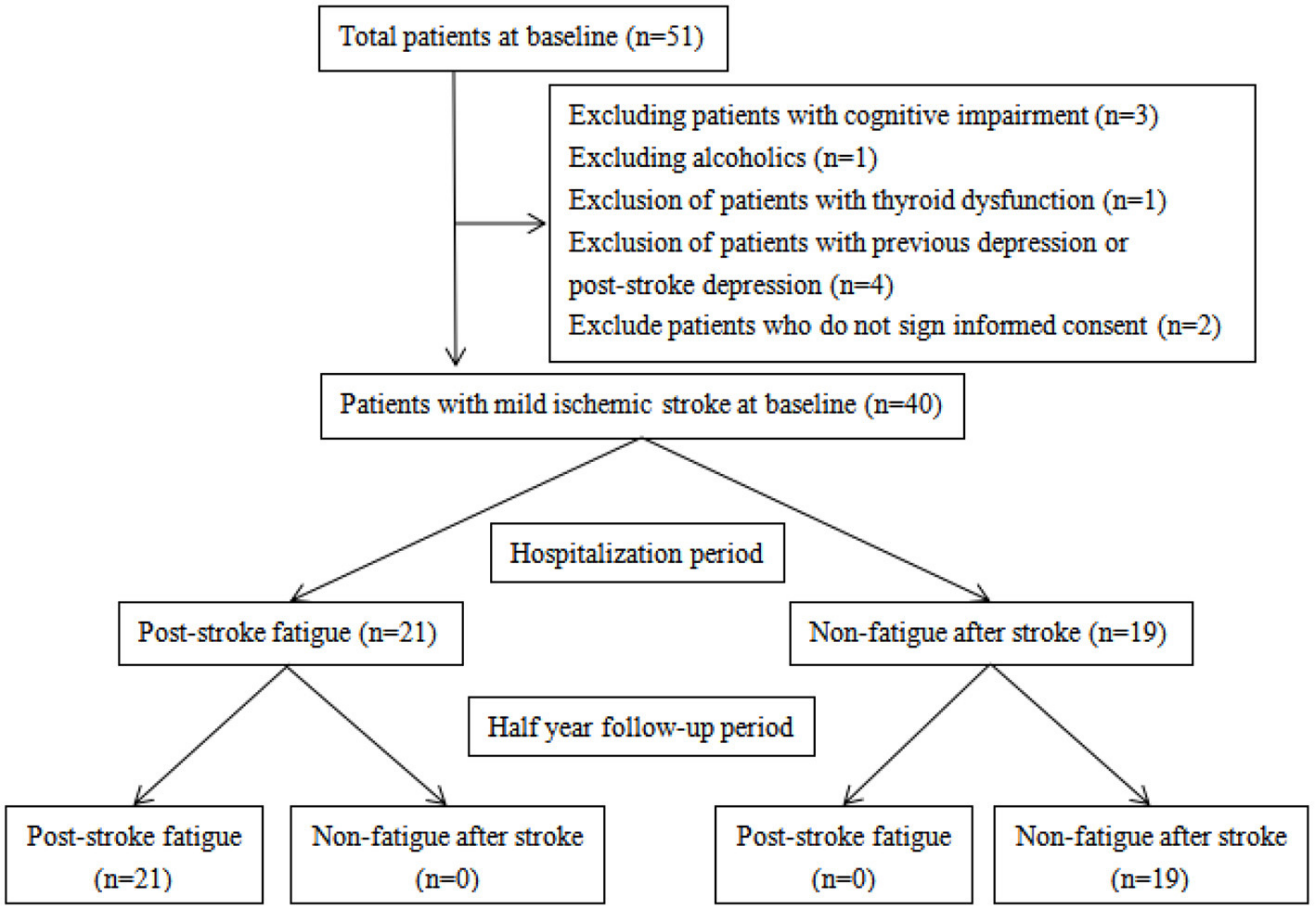
Flow chart showing patient enrollment.

A total of 40 consecutive patients were enrolled between March and June 2018. All patients have experienced mild ischemic stroke and were admitted to the Department of Neurology of Beijing Tiantan Hospital within 14 ± 2 days from onset of symptoms (Figure 1). The diagnosis of cerebral infarction was based on the WHO diagnostic criteria (including patients with first episode and relapse). All participants had NHISS ≤ 4 points and showed no obvious cognitive dysfunction.

Patients with any of the following were excluded: (1) Illiteracy and cognitive dysfunction (MOCA scale <26 points); (2) Had fatigue symptom more than 2 weeks before the onset of stroke; (3) Alcohol or drug abuse; (4) Long-term use of drugs for mental illness; (5) With central nervous system disease or thyroid disease other than cerebrovascular disease; (6) Cannot complete the examination due to hearing, vision, language expression, disturbance of consciousness, or understanding problems; (7) With severe organ dysfunction, malignancy, or life expectancy of <6 months; (8) With poor compliance or difficulty in completing follow-up; (9) History of depression or post-stroke depression (excluded using the MINI scale).

This study was approved by the Medical Ethics Committee of Beijing Tiantan Hospital (z16110000216131). Written informed consent was obtained from all participants or family members.

Patients were followed up at the outpatient clinic at 6 months from onset. The evaluation indexes included the depression scale, Pittsburgh sleep scale, fatigue-14 scale, fatigue severity scale, modified Rankin scale (mRS), and NIHSS scores.

The prognosis of brain functional status after stroke was measured with the mRS score, and the prognosis was defined as good prognosis (0–2 points) and poor prognosis (3–6 points). Scale evaluation was performed by two independent trained researchers who did not have access to final data processing and statistical analysis.

### Data Collection

#### Clinical Data Collection

Demographic data (age, gender, height, and weight) were recorded upon admission. The following stroke risk factors were assessed: anemia, previous hypertension, diabetes, coronary heart disease, hypoalbuminemia, atherosclerotic stroke, smoking, drinking, and snoring history. Types of TOAST (Trial of Org 10172 in Acute Stroke Treatment) criteria included large-artery atherosclerosis (LAA), small-artery occlusion (SAO), cardiac embolism (CE), and other determined and undetermined pathogenesis.

#### Collection of Peripheral Blood Biology Information

The following Biopike kits (manufacturer, City, State or Country) were used: serotonin (5-HT) detection kit (CEA808Ge 96T), dopamine (DA) detection kit (CEA851Ge 96T), and noradrenaline (NE) detection kit (CEA907Ge 96T). Cryopreserved serum specimens were shipped with dry ice to the kit manufacturer for post-processing. Kit instructions were rigorously followed during steps of reagent preparation, sample addition, incubation, washing, and concentration. The kits use competitive inhibition enzyme-linked immunosorbent assay to determine the level of the test substance in the specimen.

#### Head Magnetic Resonance Data Acquisition

All patients completed a head magnetic resonance scan with Philips ingenia 3.0t (manufacturer, City, State or Country) during hospitalization, and 16 had a functional MRI scan. Scan sequences include 3DT1 (Layer thickness 1 mm, layer number 196, no interval, TR 6.7 ms, TE 3.0 ms, FOV 256 × 256 × 196 mm), T2FLAIR (Layer thickness 1 mm, layer number 196, no interval, TR 4800 ms, TI 1650 ms, TE 207 ms, FOV 256 × 256 × 196 mm), DWI, ADC, MRA (Layer thickness 1.4 mm, layer number 116, layer interval −0.7 mm, TR 23 ms, TE 3.5 ms, FOV 200 × 200 × 81 mm), and resting state of functional magnetic resonance (Layer thickness 4 mm, layer number 40, no interval, TR 2000 ms, TE 30 ms, FOV 240 × 240 × 160 mm, lasting for 6 min and 24 s). The peak coordinates of different brain regions as the center of the ball were taken, and the radius was 3 voxels. Each sample value was extracted.

Functional MRI post-processing was performed using SPM and Rest software (manufacturer, City, State or Country), and the following parameters were applied: format conversion, removal of the first 10 time points, time layer correction (Session 170 files, Number of Slices 40, TR 2, TA 1.95, Slice order 1 × 40 double, Reference Slice 39, Filename Prefix a), head movement correction (Session 170 files, Quality 0.9, Separation 4, Smoothing 5), spatial standardization (Bounding box 2 × 3 double, Voxel sizes [2 2 2]), spatial smoothing (images to smooth 170 files, FWHM [8 8 8]), and other post-processing operations. Low frequency fluctuations (ALFF), Regional homogeneity (ReHo), and Functional connectivity (FC) were calculated directly by the software. Reports of post-processing were reviewed and issued by radiologists.

ALFF reflects the average intensity of the low frequency part of each voxel BOLD signal, representing the spontaneous activity of neurons in the resting state. ReHo measures the consistency of a voxel and its surrounding voxels, which is calculated based on Kendall coefficient of concordance (KCC) and represents the activity consistency of adjacent neurons in resting state. The higher the ReHo value, the better the consistency between local voxels and neighboring voxels, but it does not necessarily mean the more significant the local neural activity. FC represents the synergy of spontaneous activity of neurons in resting state.

### Definition of Post-stroke Fatigue

We adopted the 2007 Lynch case definition (17) of hospitalized patients in this study: the patient has experienced fatigue and lack of energy daily or almost daily following the stroke onset, which leads to difficulties in participating in daily activities. The fatigue severity scale (FSS) was used to quantify fatigue as follows: no fatigue (<36 points) and fatigue (≥36 points). The Fatigue-14 Scale was used to assess the severity of fatigue. Higher scores indicate more severe fatigue.

### Statistical Analysis

Statistical analysis was performed using SPSS 24.0 (manufacturer, City, State or Country). Continuous variables were tested for normal distribution. Student *t*-test and rank sum test were used to compare normally and abnormally distributed continuous variables, respectively. Categorical data were tested by chi-square test. Univariable analysis was used to assess variables associated with post-stroke fatigue. Variables with a univariable *P* ≤ 0.1 were entered into a multivariable logistic regression model of fatigue after mild ischemic stroke. Candidate variables included patient age, gender, presence of hypertension, PSQI score during hospitalization, and blood levels of three biological markers. All tests were two-sided, and a *P* < 0.05 was considered statistically significant.

## Result

### Baseline Information

Among 40 patients with mild ischemic, mean age was 59.3 ± 10.7 years (range 34–78), 34 were male, and 21 patients (52.5%) were diagnosed with fatigue after mild ischemic stroke. Of the 34 male patients, 17 patients (50%) had post mild-ischemic-stroke fatigue, and 17 (50%) did not (Table 1). The gender, age, height, weight, BMI value, diabetes, heart failure, proteinuria, hypoalbuminemia, anemia, smoking, drinking, snoring, whether it is aortic atherosclerotic stroke, blood dopamine level, mRs score, and NIHSS score during hospitalization did not differ significantly patients with and without fatigue after mild ischemic stroke (*P* > 0.1). Compared with the non-fatigue group, patients in the post-stroke fatigue group had higher PSQI scores during hospitalization (10.5 ± 5.3 vs. 7.6 ± 5.0, *p* = 0.047) (Table 1).

**Table 1:** Demographic and clinical variables during hospitalization and follow-up.

	**Total (*n* = 40)**	**Fatigue after ischemic stroke**		***P*-value**
		**Yes (*n* = 21)**	**No (*n* = 19)**	
Male	34 (85)	17 (81)	17 (89.5)	0.451
Age	59.3 ± 10.7	56.6 ± 9.9	61.7 ± 11.1	0.122
Height (cm)	168.8 ± 4.8	167.9 ± 4.5	169.8 ± 5.1	0.111
Weight (kg)	71.2 ± 10.0	68.8 ± 8.4	73.7 ± 11.2	0.121
Body mass index (kg/m^2^)	24.9 ± 2.7	24.3 ± 2.2	25.5 ± 3.1	0.184
Hypertension	28 (70)	17 (81)	11 (57.9)	0.100
Diabetes	16 (40)	9 (42.9)	7 (36.8)	0.698
Heart failure	10 (25)	7 (33.3)	3 (15.8)	0.201
Proteinuria	8 (20)	5 (23.8)	2 (15.8)	0.527
Hypoalbuminemia	2 (5)	2 (9.5)	0 (0)	0.168
Anemia	2 (5)	2 (9.5)	0 (0)	0.168
Atherosclerotic stroke	32 (80)	16 (76.2)	16 (84.2)	0.527
Smoking	25 (62.5)	11 (52.4)	14 (73.7)	0.165
Drinking	23 (57.5)	11 (52.4)	12 (63.2)	0.491
Snore	18 (45)	11 (52.4)	17 (36.8)	0.324
Poor prognosis during hospitalization^†^	10 (25)	7 (33.3)	3 (15.8)	0.201
Anterior circulation infarction	20 (50)	10 (47.6)	10 (52.6)	0.752
Striatum-thalamus-frontal cortex involvement	27 (67.5)	17 (81)	10 (52.6)	0.056
Cerebellar infarction	7 (17.5)	5 (23.8)	2 (10.5)	0.270
Brain stem infarction	16 (40)	10 (47.6)	6 (31.6)	0.301
Right cerebral hemisphere infarction	23 (57.5)	17 (81)	6 (31.6)	0.002
Frontal lobe infarction	5 (12.5)	3 (14.3)	2 (10.5)	0.720
Parietal lobe infarction	5 (12.5)	3 (14.3)	2 (10.5)	0.720
Temporal lobe infarction	19 (47.5)	9 (42.9)	10 (52.6)	0.536
Occipital lobe infarction	2 (5)	2 (9.5)	0 (0)	0.168
Insular infarction	7 (17.5)	5 (23.8)	2 (10.5)	0.270
NIHSS during hospitalization	2.2 ± 0.9	2.2 ± 1.0	2.2 ± 1.0	0.848
NIHSS during follow-up	1.4 ± 0.5	1.4 ± 0.6	1.3 ± 0.5	0.367
Total PSQI score in hospitalization^‡^	9.2 ± 5.3	10.5 ± 5.3	7.6 ± 5.0	0.047
Total PSQI score during follow-up	8.5 ± 4.7	10.1 ± 4.9	6.7 ± 3.8	0.023
Norepinephrine levels (pg/ml)	0.5 ± 0.1	0.4 ± 0.1	0.6 ± 0.1	<0.001
Dopamine levels (pg/ml)	0.9 ± 0.2	0.9 ± 0.2	0.9 ± 0.2	0.715
Serotonin levels (ng/ml)	1.4 ± 0.3	1.3 ± 0.3	1.5 ± 0.3	0.026

† *Define good prognosis (0–2 points) and poor prognosis (3–6 points) according to mRs score*.

‡ *PSQI, Pittsburgh Sleep Quality Index scale total score*.

After 6 months of follow-up, all patients in the post-stroke fatigue group remain in fatigue, and no fatigue symptoms were occurring in non-post-stroke fatigue patients. Moreover, cognitive and depression measured by MINI and MOCA scales showed no changes in all 40 patients. No patients of two groups showed cognitive decline or depression.

### Correlation Between the Monoamine Levels and Severity of Fatigue

**Figure 2:**
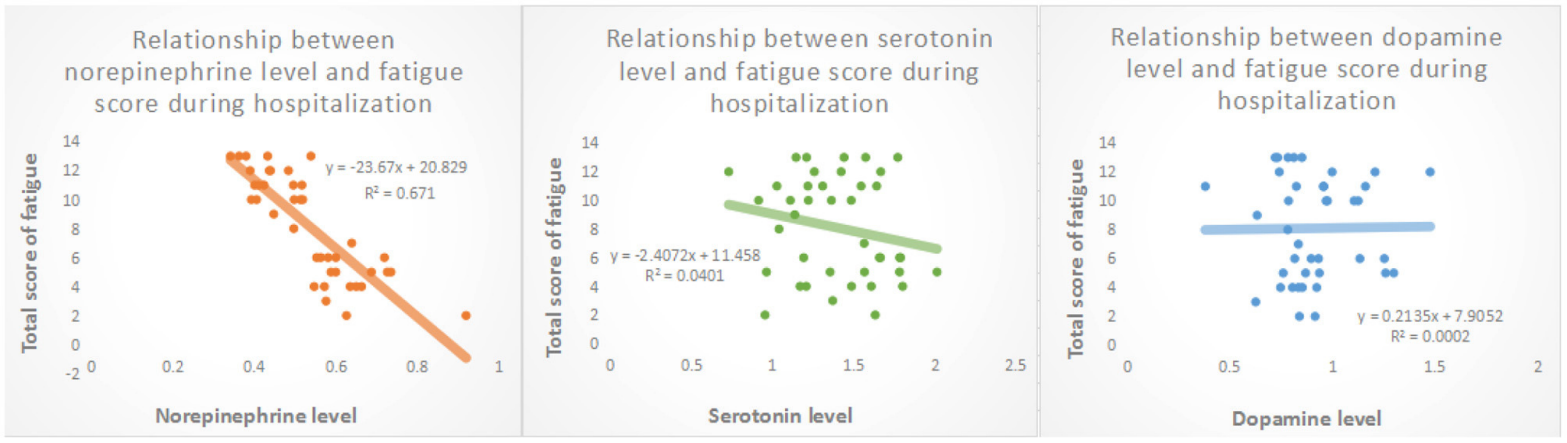
Relationship between levels of biological transmitters in peripheral venous blood and fatigue scores during hospitalization.

Compared to non-fatigue group, blood norepinephrine and serotonin levels (0.444 ± 0.058 pg/ml, *P* < 0.001 and 1.297 ± 0.256 ng/ml, *p* = 0.026, respectively), in the post-stroke fatigue group were lower during the hospitalization period (Table 1). Figure 2 shows that the norepinephrine and serotonin levels during hospitalization were negatively correlated to the Fatigue-14 scale score. However, no correlation between the blood dopamine level and the severity of fatigue during hospitalization was observed.

As shown in Table 2, patient age was positively correlated with the prevalence of fatigue (*p* = 0.009). Both norepinephrine and serotonin levels during hospitalization were negatively correlated to the prevalence of post-stroke fatigue (model 1 *p* = 0.009 and model 2 *P* = 0.043, respectively). The gender, presence of hypertension, PSQI score during hospitalization, and dopamine level during hospitalization were not associated with post-stroke fatigue (*P* > 0.05).

**Table 2:** Multivariable analysis of fatigue after mild ischemic stroke during hospitalization.

	**Odds ratio (95% confidence interval)**	***P***
**Model 1**		
Male	4.0 (0.2–87.1)	0.376
Age	1.2 (1.0–1.3)	0.009
PSQI score during hospitalization	1.3 (1.0–1.6)	0.094
Hypertension	8.8 (0.6–130.5)	0.114
Norepinephrine levels during hospitalization	0.0 (0.0–0.1)	0.009
**Model 2**		
Male	0.8 (0.1–6.4)	0.817
Age	1.1 (1.0–1.1)	0.043
PSQI score during hospitalization	4.2 (0.8–23.7)	0.099
Hypertension	1.1 (1.0–1.3)	0.192
Serotonin levels during hospitalization	0.1 (0.0–0.4)	0.011
**Model 3**		
Male	0.3 (0.1–1.7)	0.167
Age	1.0 (1.0–1.1)	0.467
PSQI score during hospitalization	2.1 (0.5–9.2)	0.321
Hypertension	1.1 (1.0–1.2)	0.218
Dopamine levels during hospitalization	0.3 (0.1–5.3)	0.411

### Imaging Mechanism of Fatigue After Mild Ischemic Stroke Onset

Compared to non-fatigue group, the post-stroke fatigue group were more likely to have right cerebral hemisphere infarction (81%, *p* = 0.002), and a higher PSQI score during hospitalization (10.6 ± 5.3, *p* = 0.047). There was no significant difference in infarction of the anterior and posterior circulation, the frontal, parietal and temporal lobes, insula, occipital lobe, brain stem, and cerebellum between two groups. Infarct of right cerebral hemisphere was positively correlated to the occurrence of fatigue after mild ischemic stroke (*p* = 0.020) (Table 1). However, gender, age, hypertension, PSQI score, and involvement of the striatum-thalamus-frontal cortex were not associated with the incidence of fatigue after mild ischemic stroke (*P* > 0.05) (Table 3).

**Table 3:** Multivariable analysis of fatigue after mild ischemic stroke.

	**Odds ratio (95% confidence interval)**	***P*-value**
Males	0.2 (0.0–1.2)	0.078
Age	1.0 (0.9–1.0)	0.485
Striatum-thalamus-frontal cortex circuit involvement	1.0 (0.1–8.6)	0.971
Right cerebral hemisphere infarction	10.0 (1.4–70.4)	0.020
Hypertension	2.3 (0.4–12.9)	0.330
PSQI score	1.1 (0.9–1.3)	0.318

**Figure 3:**
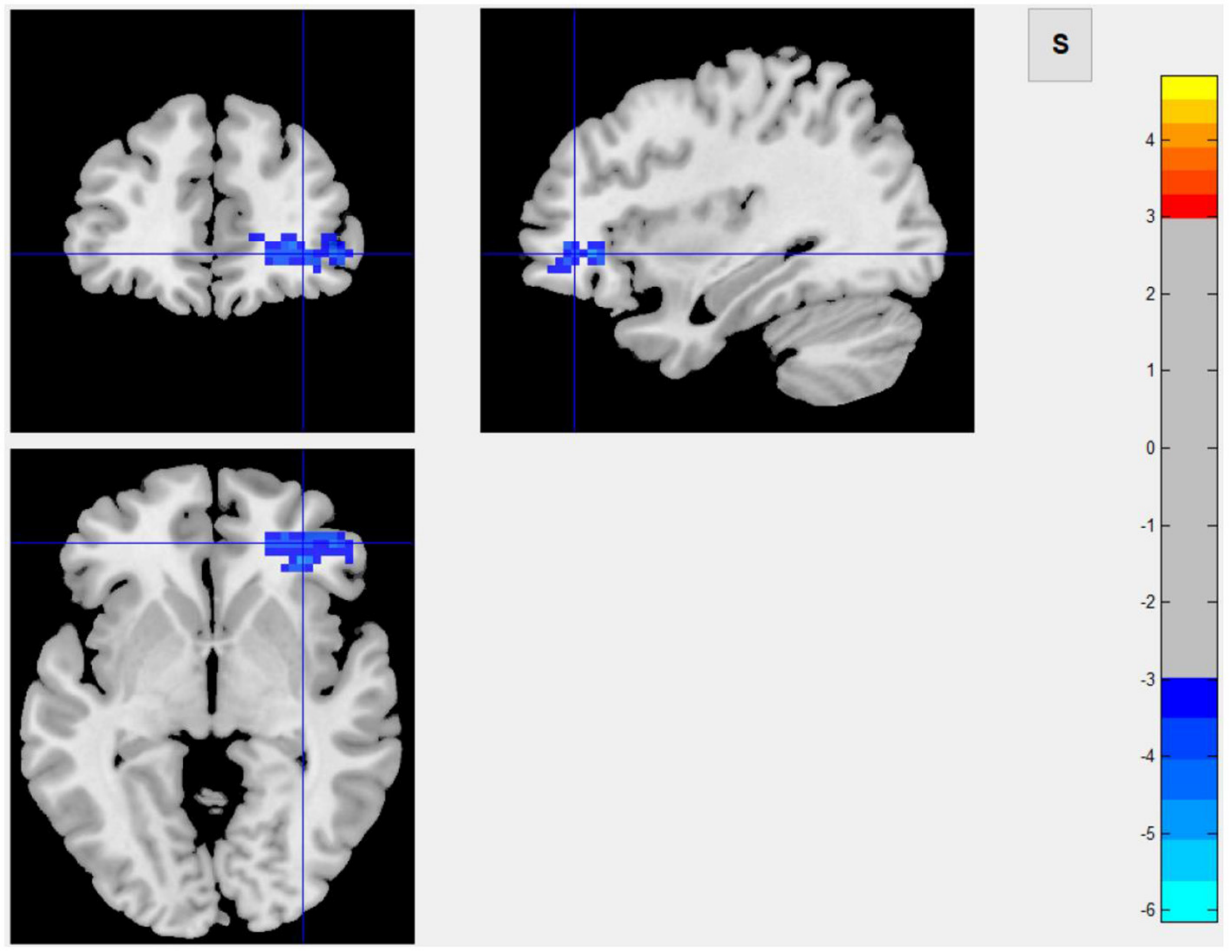
Area of ALFF reduction in the post-stroke fatigue group compared to the non-fatigue group.

**Figure 4:**
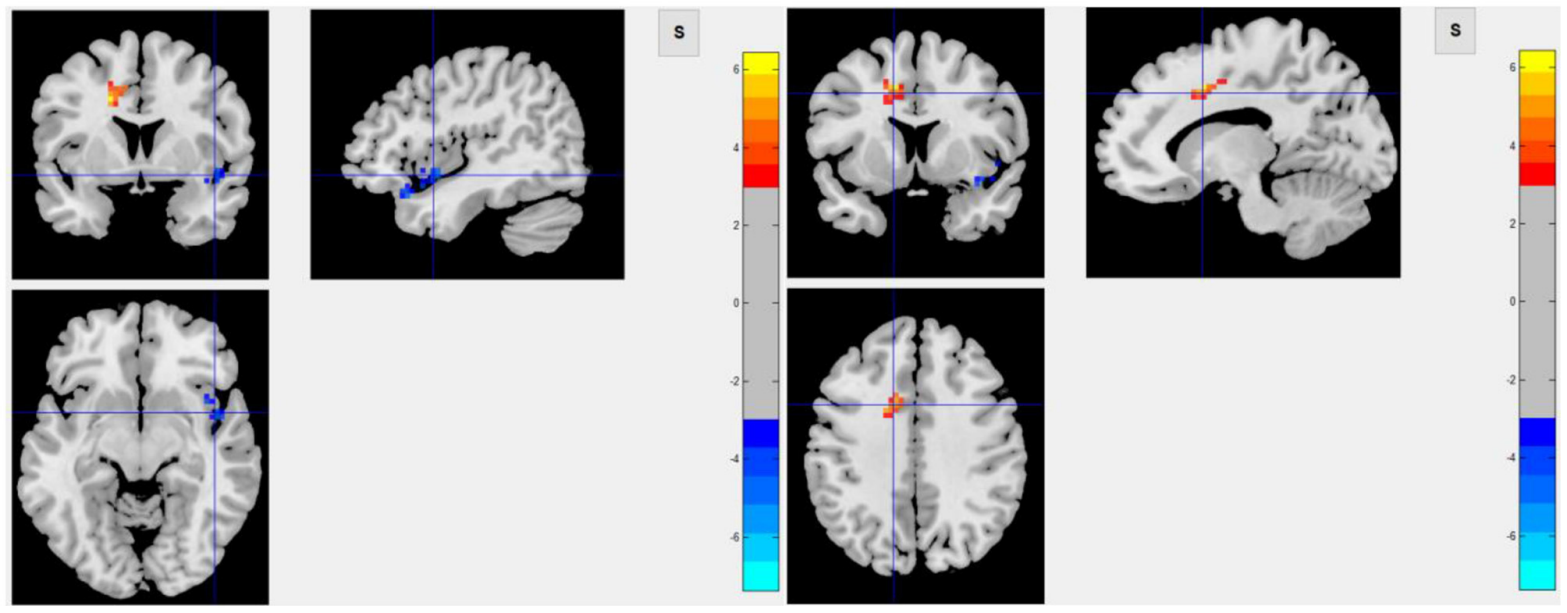
The left panel shows the area of decrease of ReHo value in the post-stroke fatigue group compared to the non-fatigue group, and the right panel shows the area of increase of ReHo value in the post-stroke fatigue group compared to the non-fatigue group.

Among 16 patients with functional MRI scans, 9 had post-stroke fatigue and 7 did not. Compared to non-fatigue patients, ALFF values were lower in several areas of the brain in stroke patients with fatigue, including the right orbital inferior frontal, right inner orbital frontal, right frontal, right triangular frontal inferior, right anterior and lateral cingulate, and the right medial frontal gyruses (Figure 3 and Table 4). ReHo values were lower in some areas of post-stroke fatigue patient brain, including the right superior temporal gyrus, the right insula, and the right orbital inferior frontal gyrus. ReHo values were higher in other brain areas of the post-stroke fatigue group, including the left medial and lateral cingulate gyrus, the left supplementary exercise area, and the left dorsolateral frontal gyrus (Figure 4 and Table 4). No cluster was found in the functional connectivity between the fatigue and non-fatigue groups.

**Table 4:** Alff and ReHo values in different brain regions in patients with and without post-stroke fatigue.

	**Post-stroke fatigue (*n* = 9)**	**Post-stroke non-fatigue (*n* = 7)**	***T***	***P*-value**
ALFF	0.7 ± 0.1	0.8 ± 0.2	−4.7	<0.01
ReHo 1	0.6 ± 0.2	0.7 ± 0.1	−7.4	<0.01
ReHo 2	1.1 ± 0.3	0.9 ± 0.1	6.5	<0.01

*ReHo 1: Brain regions with reduced ReHo value, corresponding to the blue area in Figure 4*.

*ReHo 2: Brain regions with increased ReHo value, corresponding to the orange area in Figure 4*.

## Discussion

Few studies have investigated the biological markers and imaging mechanisms of post-stroke fatigue in the population of China. Our study has found that age and norepinephrine and serotonin levels are risk factors of fatigue after mild ischemic stroke. The pathogenesis of post mild-ischemic-stroke fatigue may be that stroke affects non major hemispheric related neural pathways, especially frontal lobe related neural pathways, resulting in a decrease of neurotransmitters, such as norepinephrine and 5-hydroxytryptamine, which leads to post-stroke fatigue.

Due to the lack of a unified definition for post-stroke fatigue, different definitions and evaluation methods have been used, and the morbidity and prevalence rates were various in different studies. Cumming and associates reported an estimated prevalence of post-stroke fatigue of 25–85% (18), while the incidence was between 35 and 92% in another study (19). Our study found an incidence of 52.5% (21 out of 40), which is in line with previous studies.

The disease duration of post-stroke fatigue was also inconclusive. Some studies have suggested that post-stroke fatigue symptoms may last for a long time (19, 20). In contrast, in a longitudinal cohort study Duncan et al. and Chen and Marsh showed that symptoms of post-stroke fatigue disappeared or greatly eased within 12 months after stroke onset (21, 22). Our study found that post-stroke fatigue symptoms and their effects on daily life persisted at least for 6 months after stroke onset. However, due to the small sample size and some potential selection bias, further study in a large sample size is warranted. We did not find any correlation between the occurrence of fatigue after mild ischemic stroke and poor prognosis of stroke. This may be due to the mild stroke with NIHSS score of 4 or less in enrolled patients and the follow-up duration of 6 months, which is the best recovery period after stroke. With the recovery of neurological function, the mRS scores decreased correspondingly, which may interfere with the influence of fatigue on the prognosis.

So far, few studies have investigated demographic risk factors of post-stroke fatigue, and the findings of existing studies are contradictory. While some studies show that post-stroke fatigue was independently associated with pre-stroke depression, cognition, leukocytosis, myocardial infarction, diabetes, pain, and sleep disorders (7, 19, 20, 23–26), others find that variables such as gender, marital status, education level, emotional state, excessive daytime sleepiness, waist circumference, hypertension, ischemic heart disease, atrial fibrillation, and diabetes were not significantly associated with the occurrence of post-stroke fatigue (8, 27, 28). In this study, patients did not have depression and cognitive decline even after 6 months, which suggested that post-stroke fatigue symptoms were not caused by depression and cognitive impairment. Age and PSQI score at 14 ± 2 days of mild ischemic stroke onset were identified as risk factors of fatigue, which may be explained by the small sample size of this study.

Concerning the biological mechanisms of post-stroke fatigue, some studies proposed that the immune response after ischemic stroke may predict the occurrence of post-stroke fatigue (29, 30). In Korean patients, it was found that post-stroke fatigue may be associated with genetic polymorphisms within the MAO-A gene in females (31). Hama et al. showed that the monoamine neural network can be divided into catecholamine and serotonin branches, which are related to each other anatomically and functionally, and may affect the development of post-stroke fatigue and emotional disorders (14). However, a systematic review did not find a clear correlation between hypothalamic-pituitary-adrenal axis disorder or neurotransmitter changes and post-stroke fatigue (32). In the present study, the norepinephrine and the serotonin levels, rather than the dopamine level, were negatively correlated with the severity of post-stroke fatigue, which implies that abnormal monoamine neurotransmitter circuits may be at play in the pathogenesis mechanism of post-stroke fatigue.

The Bergen Stroke Study found that the average FSS score of patients with TIA was lower than those with mild cerebral infarction, suggesting that ischemic stroke may cause some brain tissue damage and lead to post-stroke fatigue in some patients (7). This finding has led researchers to study the imaging mechanisms of post-stroke fatigue, but results were mixed. The relationship is unclear between the occurrence of post-stroke fatigue and the characteristics of stroke, such as location, type and number of strokes, and neurological deficits (16). Chaudhuri and Behan have shown that central fatigue may be caused by damages to the striatum-thalamo-frontal cortex system due to the failure of integration of peripheral input and basal ganglia motor function (15). Other studies found an association between post-stroke fatigue and subatentorial injury sites (particularly the brain stem) or basal ganglia stroke (3, 15, 33, 34). These results suggest that the disruption of neural networks, which regulates the nervous attention such as the reticular activation system, may be a key risk factor for fatigue. While post-stroke fatigue symptoms were reported to be more severe in cortical excitability deficiency human (35), the area, location, and atrophy of cerebral infarction were not associated with the prevalence of fatigue after ischemic stroke (36).

In this study, infarct of the right cerebral hemisphere was significantly correlated to the occurrence of fatigue after mild ischemic stroke, and such patients had a lower degree of spontaneous neuronal activity in the right orbital inferior frontal, right inner orbital frontal, right middle frontal, right triangular frontal inferior, right anterior and lateral gyrus, and right medial frontal gyruses, as well as a lower ReHo Score in the right superior temporal gyrus, right insula, and right orbital inferior frontal gyrus. These results suggested that the consistency of adjacent neurons was lower in post-stroke fatigue patients. Since the 40 patients enrolled in this study are all right-handed, the right cerebral hemisphere was a non-dominant hemisphere. In theory, the striatum, thalamus, and subfrontal cortex loop in the non-dominant hemisphere are related to a variety of emotional and mental activities. Because fatigue is considered as a type of mental and emotional activity, it is largely related to the aforementioned cerebral structures. The observed decrease in the activity of frontal lobe neurons in the non-dominant hemisphere in post-stroke fatigue patients indicates the post-stroke fatigue may lower the activity of frontal lobe neurons. Although no changes of morphology in the striatum and thalamus were observed, the decreased activity of frontal lobe neurons could be ascribed to the damage of conduction loop in the striatum and thalamus, which cannot be observed. According to the central fatigue neurotransmitter hypothesis, after prolonged activity, the synthesis and metabolism of monoamine neurotransmitters in the brain, such as serotonin, dopamine, and norepinephrine, will change and may lead to an increase in sleep duration and lack of motivation, which causes a feeling of tiredness (37). In the left medial and lateral cingulate gyruses, the left supplementary motor area, and the left dorsolateral frontal gyrus, the activity of adjacent neurons in the post-stroke fatigue group was consistent.

Based on previous finding that fatigue after stroke is a central fatigue (38), our hypothesis on the pathogenesis of fatigue after mild ischemic stroke assumes that stroke damages the non-dominant hemisphere-related neural pathways, especially the frontal lobe-related neural pathways. These damages result in a decrease of neurotransmitters such as norepinephrine and serotonin, which leads to post-stroke fatigue. Unlike depression after stroke, post-stroke fatigue is more likely to be caused by the destruction of the norepinephrine circuit, rather than by the abnormal dopamine circuit.

This study has several limitations, such as the single-center setting, which may not reflect the overall situation of the total population in China. The small sample size may lead to an increased risk of sampling error, resulting in false positive or false negative results and affecting the reliability of the conclusion. In addition, some patients were not included in the study due to bed constraints and other factors, and some were excluded as they were discharged from the hospital 14 days after onset or could not complete blood collection, which may involve a selection bias.

## Conclusion

Frontal lobe-related neural pathways may play an essential role in the regulation of fatigue after mild ischemic stroke. Abnormal neural circuits may reduce neurotransmitters such as serotonin and norepinephrine, leading to post-stroke fatigue.

## Data Availability Statement

The raw data supporting the conclusions of this article will be made available by the authors, without undue reservation.

## Ethics Statement

The studies involving human participants were reviewed and approved by Medical Ethics Committee of Beijing Tiantan Hospital (z16110000216131). The patients/participants provided their written informed consent to participate in this study.

## Author Contributions

XZ: data collection, conceptual research, statistical analysis, result interpretation, and manuscript writing. HF: revision of the manuscript. DM and YD: image scanning. ZW: post-processing of functional magnetic resonance. NZ and CW: conception and revision of the manuscript. All authors contributed to the article and approved the submitted version.

## Conflict of Interest

The authors declare that the research was conducted in the absence of any commercial or financial relationships that could be construed as a potential conflict of interest.
